# Up-regulation of SPC25 promotes breast cancer

**DOI:** 10.18632/aging.102153

**Published:** 2019-08-10

**Authors:** Qian Wang, Yanhui Zhu, Zhouxiao Li, Qian Bu, Tong Sun, Hanjin Wang, Handong Sun, Xiufeng Cao

**Affiliations:** 1 Department of Oncology Surgery, Nanjing First Hospital, Nanjing Medical University, Nanjing, China; 2 Department of Breast Surgery, The First Affiliated Hospital of Nanjing Medical University, Nanjing, China; 3 Department of Hand Surgery, Plastic Surgery and Aesthetic Surgery, Ludwig-Maximilians University, Munich, Germany; 4 Department of Medical Oncology, Taizhou People’s Hospital, Jiangsu University, Zhenjiang, China; 5 Key Laboratory of Human Functional Genomics of Jiangsu Province, Nanjing Medical University, Nanjing, China; 6 Department of General Surgery, Nanjing First Hospital, Nanjing Medical University, Nanjing, China; 7 Department of Thoracic Surgery, Taikang Xianlin Drum Tower Hospital, Nanjing University, Nanjing, China

**Keywords:** SPC25, data mining, immune, proliferation, DNA methylation

## Abstract

In this study, expression of the SPC25 gene was characterized in breast cancer (BC), and its effects on BC development and progression, functions in BC cells, and potential underlying mechanisms were examined. Data from TCGAportal and FIREBROWSE indicated that SPC25 was upregulated in BC tissues compared to normal tissues, and CANCERTOOL indicated that higher SPC25 mRNA levels were associated with increased probability of recurrence and poorer survival in BC patients. BC patients with higher SPC25 expression displayed shorter distant metastasis-free survival, relapse-free survival, and overall survival. Colony formation and CCK-8 experiments confirmed that SPC25 promoted proliferation of BC cells. Single-cell analysis indicated that SPC25 is associated with cell cycle regulation, DNA damage and repair, and BC cell proliferation. SPC25 knockdown suppressed proliferation of BC cells. MiRNAs, circRNAs, RNA-binding proteins, transcription factors, and immune factors that might interact with SPC25 mRNA to promote BC were also identified. These findings suggest that SPC25 levels are higher in more malignant BC subtypes and are associated with poor prognosis in BC patients. In addition, DNA methyltransferase inhibitor and transcription factors inhibitor treatments targeting SPC25 might improve survival in BC patients.

## INTRODUCTION

Breast cancer (BC) is one of the most common malignancies in women, and occurrences in young women are increasing [1–2]. Despite significant advances in the diagnosis and treatment of tumors in recent years, survival times remain short for some BC patients. BC is a heterogeneous disease that includes multiple subgroups with different molecular characteristics, prognoses, and therapeutic responses [3]. Clinically, BC can be subdivided into three major subtypes: estrogen (ER) and/or progesterone receptor (PgR) expressing tumors (commonly referred to as hormone receptor positive [HR+]), ErbB2 amplification [HER2+]), and triple-negative BC (TNBC), in which ER/PgR expression is normal and HER2 expression is normal or lacking [4]. Identification of novel effective biomarkers for diagnosing BC and assessing prognosis would aid in treatment.

Spindle component 25 (SPC25) is a protein involved in kinetochore-microtubule interactions and spindle checkpoint activity. Tumorigenesis can arise from genetic instability in the cell cycle resulting from inaccurate chromosomal segregation, a process in which kinetochores play a crucial role [5]. A recent study reported that SPC25 is up-regulated in lung cancer and is associated with carcinogenesis, cancer cell growth, and metastasis [6]. SPC25 expression is also elevated in prostate cancer (PrC) and affects proliferation and cell cycle progression in PrC cells [7–8]. Pathania and colleagues reported that SPC25 is significantly overexpressed in human breast tumor tissues and is associated with reduced overall survival in BC patients [9]. However, the detailed functions and molecular mechanisms of SPC25 in BC remain unknown.

In the present study, therefore, we examined the expression, molecular mechanisms, and clinical relevance of SPC25 in BC.

## RESULTS

### *SPC25* is over-expressed in BC

**Figure 1:**
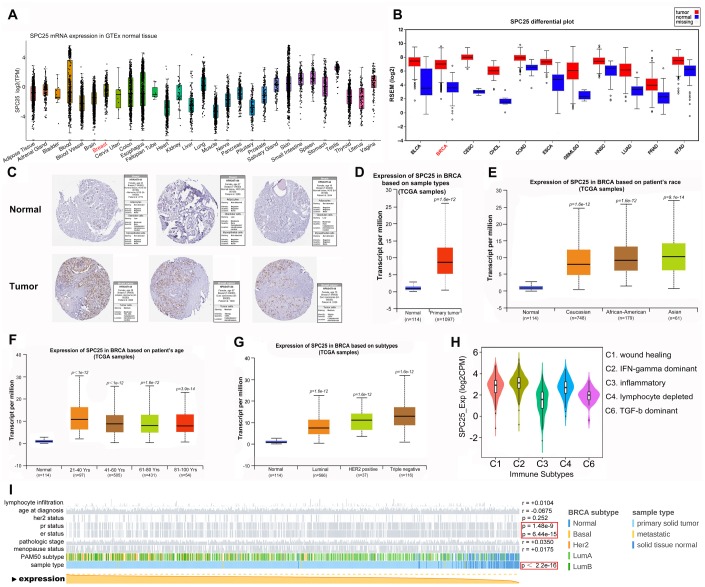
**SPC25 expression overview.** (**A**) SPC25 mRNA expression in normal tissues. (**B**) SPC25 mRNA expression in human cancers is significantly higher than in normal tissues. (**C**) SPC25 expression in normal breast tissues and BC tissues. (**D**) SPC25 mRNA expression is significantly higher in BC samples than in normal samples. (**E**–**H**) Differences in SPC25 mRNA expression depending on race, age, molecular subtype, and immune subtype. (**I**) SPC25 mRNA expression is correlated with PR status, ER status, and sample type.

We initially examined *SPC25* expression in normal and BC tissues using GTEx and FIREBROWSE. *SPC25* expression, which was present in most human tissues (Figure 1A), was significantly higher in BC tumor tissues than that in corresponding normal tissues (Figure 1B). Analysis of Human Protein Atlas data indicated that SPC25 staining is stronger in BC tissue than in normal breast tissue (Figure 1C). Next, we performed a more comprehensive analysis of SPC25 mRNA expression in BC using UALCAN. Subgroup analysis based on race, age, molecular subtype, and immune subtype indicated that SPC25 mRNA levels are significantly higher in BC patients than in healthy individuals (Figure 1D–1H). Notably, SPC25 expression was much higher in triple negative breast cancer (TNBC) compared to other BC subtypes. Furthermore, MEXPRESS analysis indicated that SPC25 mRNA expression is negatively correlated with progesterone receptor (PR) status, estrogen receptor (ER) status, and BC sample type (Figure 1I).

### SPC25 expression is strongly associated with clinical outcome

**Figure 2:**
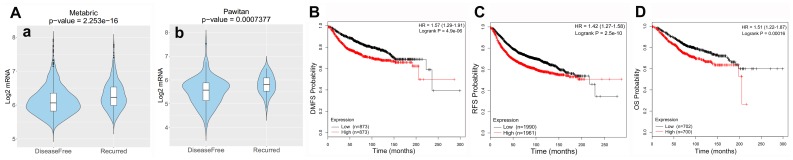
**Clinical role of SPC25 in BC patients.** (**A**) RS patients had higher SPC25 expression than DFS patients in two BC cohorts. (**B**–**D**) High SPC25 expression was correlated with poor DMFS, RFS, and OS in BC patients. (RS, survival with recurrence; DFS, disease-free survival; DMFS, distant metastasis-free survival; RFS, relapse-free survival; OS, overall survival).

The prognostic potential of SPC25 in BC was further examined using CANCERTOOL and Kaplan-Meier Plotter. Results indicated that patients with higher SPC25 expression had higher recurrence rates as well as shorter DMFS, RFS, and OS (Figure 2).

### Functions of *SPC25* in BC cells

#### SPC25 promotes cell cycle progression, DNA damage and repair, and proliferation in BC

**Figure 3:**
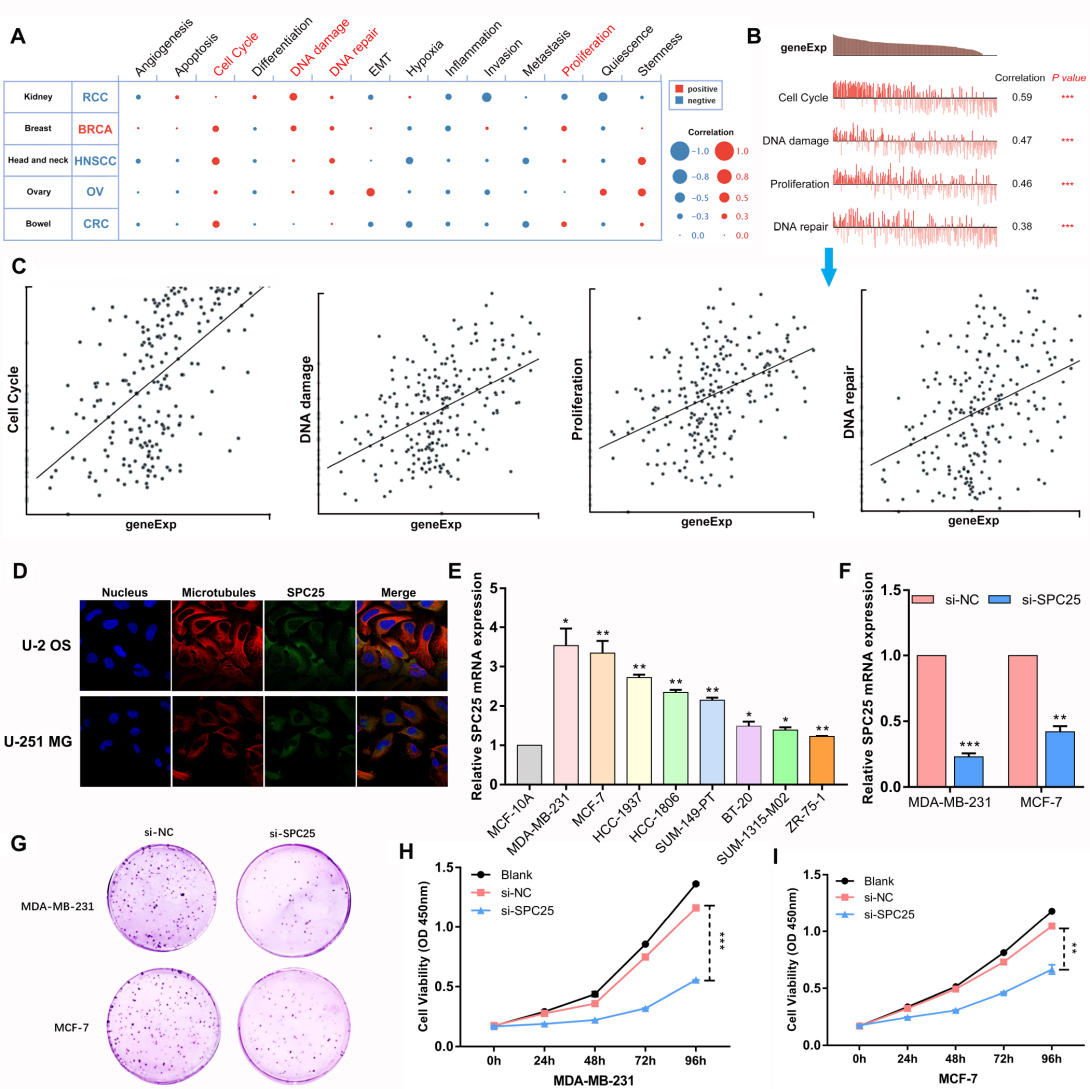
**The function of SPC25 in BC cells.** (**A**) Single-cell analysis indicated that SPC25 is primarily involved in regulation of the cell cycle, DNA damage and repair, and proliferation in BC. (**B**, **C**) Data from Braune EB (No. cells = 369) demonstrated that SPC25 mRNA expression was positively correlated with regulation of the cell cycle, DNA damage and repair, and proliferation. (**D**) The Human Protein Atlas database revealed that SPC25 was colocalized with microtubule proteins in the cytoplasm of U-2 OS and U-251 MG cells. (**E**) SPC25 expression in BC cells. (**F**) Knockdown in MDA-MB-231 and MCF-7 cells with si-SPC25 or si-NC. (**G**) Colony formation and (**H**, **I**) CCK-8 assays demonstrated that knockdown of SPC25 suppressed proliferation of MDA-MB-231 and MCF-7 cells. (si-NC, siRNA negative control; *p<0.05, **p<0.01, ***p<0.001).

In order to investigate the functions of SPC25 in BC, single-cell analysis was performed using CancerSEA. The results indicated that SPC25 might primarily regulate cell cycle progression, DNA damage and repair, and proliferation in BC cells (Figure 3A). According to data from Braune EB (No. cells = 369), SPC25 gene expression and regulation potential for the four aforementioned biological processes were significantly positively correlated in BC (Figure 3B, 3C).

#### SPC25 knockdown suppressed proliferation of BC cells

According to the Human Protein Atlas database, SPC25 is located in the cytoplasm where microtubule proteins are present in U-2 OS and U-251 MG cells (Figure 3D). We confirmed that SPC25 mRNA expression was significantly higher in several BC cell lines than in the normal MCF-10A cell line (Figure 3E). The two BC cell lines in which SPC25 mRNA expression was highest were used in loss-of-function experiments. si-RNA targeting SPC25 and si-NC were transfected into MDA-MB-231 and MCF-7 cells; successful knockdown was confirmed through qRT-PCR (Figure 3F). Colony formation and CCK-8 assays revealed that SPC25 knockdown strongly inhibited proliferation in both BC cell lines (Figure 3G, 3H).

### Mechanistic prediction of TF, hub genes, miRNA, and circRNA for SPC25 in BC

#### SPC25 mRNA expression is positively correlated with FOXM1 and DNMT’s

**Figure 4:**
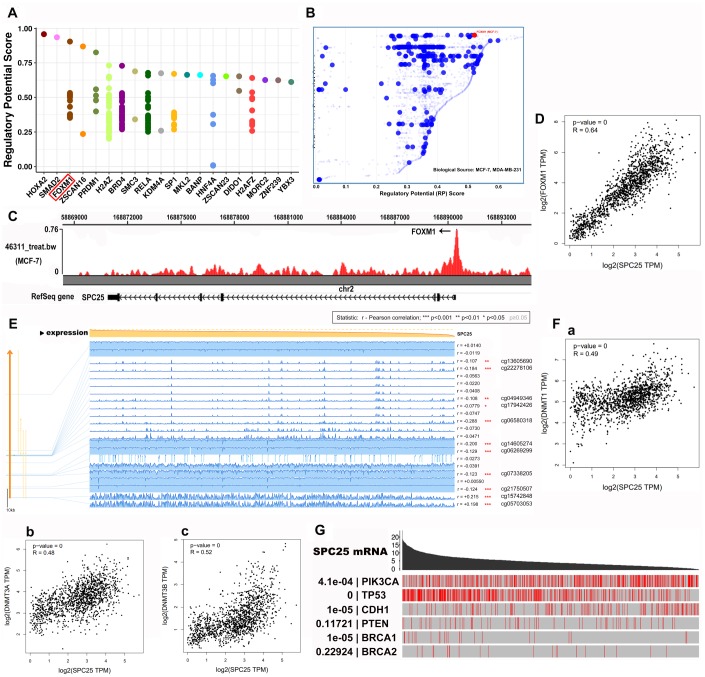
**SPC25 may regulate TFs and BC-related genes in BC.** (**A**) Top 20 TFs that potentially regulate SPC25 in different human cancers. (**B**) TFs with high regulatory potential in MDA-MB-231 and MCF-7 cell lines (10k distance to TSS). (**C**) CHIP-seq results for binding of FOXM1 and the SPC25 gene in MCF-7 cells. (**D**) Correlation between SPC25 and FOXM1 mRNA expression. (**E**) SPC25 DNA methylation modification in BC. (**F**) Correlation between SPC25 mRNA expression and DNA methyltransferase (DNMT) expression. (**G**) Correlation between SPC25 mRNA expression and mutation frequencies in tumor-associated genes. (TF, transcription factor; TSS, transcription start site. P-value=0, p<0.0001).

To identify members of a putative molecular network that regulates *SPC25* expression, we examined transcription factors (TFs) that might affect SPC25 gene transcription. First, the top 20 regulatory TFs in human cancers were identified using the Cistrome DB Toolkit (Figure 4A). Next, we specifically examined the MDA-MB-231 and MCF-7 cell lines and found that FOXM1 possessed the greatest regulatory potential in those cells (Figure 4B). Subsequent analyses revealed that binding of FOXM1 to the SPC25 gene was highest in the MCF-7 cell line and that SPC25 and FOXM mRNA expression were positively correlated in general (Figure 4C, 4D).

A previous report revealed that DNA methylation modifications play important roles in breast cancer [10]. Moreover, methylation modifications in the CpG island portion of gene promoter regions prevent some TFs from binding to DNA, thus inhibiting gene transcription. We therefore examined DNA methylation modifications in the *SPC25* gene in BC using MEXPRESS (Figure 4E). Interestingly, SPC25 and DNA methyltransferase (DNMT) expression levels were also positively correlated in BC in the GEPIA 2 database (Figure 4F). These results indicate that TFs and DNA methylation modifications might play a considerable role in BC processes by regulating *SPC25* expression.

#### Mutations in tumor-related genes interact with SPC25 to mediate BC processes

Tumor-related genes such as *PIK3CA, TP53, CDH1, PTEN, BRCA1,* and *BRCA2*, and mutations in them, play crucial roles in BC [11–15]. We therefore investigated the relationship between *SPC25* expression and mutation frequencies in these tumor-related genes in BC. As *SPC25* expression decreased, the frequency *TP53* gene mutations decreased, while the frequency of mutations in the *PIK3CA*, *CDH1*, and *BRCA1* genes increased (Figure 4G). Subsequent analysis using GEPIA 2 indicated that expression of *TP53, PIK3CA, CDH1*, and *BRCA1* was positively correlated with *SPC25* expression (Supplementary Figure 1A–D). Interestingly, expression of *MELK*, which acts as a regulatory hub gene in the TP53 pathway, was also positively correlated with *SPC25* expression (Supplementary Figure 1E).

#### miRNA, circRNA, and RBP interact with SPC25 in BC

**Figure 5:**
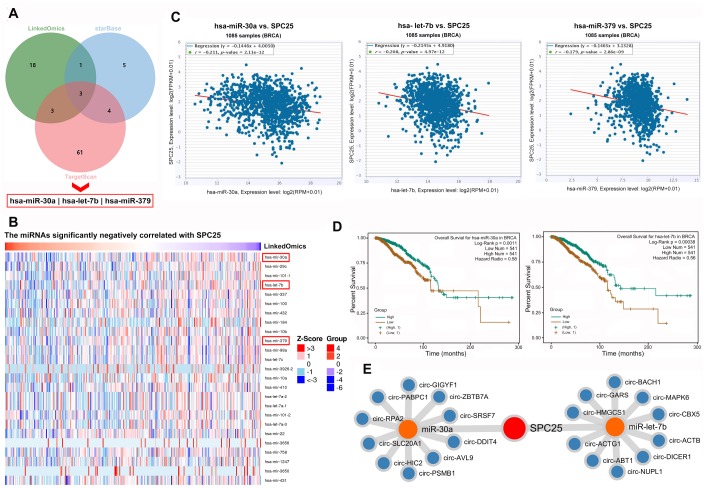
**miRNAs and circRNAs that might modulate SPC25.** (**A**) Three miRNA prediction datasets were used to select miRNAs of interest. (**B**) miRNAs negatively correlated with SPC25 mRNA expression. (**C**) starBase showed that SPC25 mRNA expression was negatively correlated with the expression of miR-30a, let-7b, and miR-379. (**D**) Patients with lower miR-30a or let-7b expression had shorter survival times. (**E**) Top 10 circRNAs interacting with miR-30a or let-7b identified by starBase.

Examination of LinkedOmics, starBase, and Targetscan, revealed three common miRNAs that were downregulated in BC according to all three databases: hsa-miR-30a, hsa-let-7b, and hsa-miR-379 (Figure 5A, 5B). Further analysis in starBase indicated that the expression of all three miRNAs was negatively correlated with *SPC25* expression in BC (Figure 5C). Most importantly, patients with higher expression of hsa-miR-30a or hsa-let-7b had shorter survival times (Figure 5D). Because circRNAs can further regulate gene expression by sponging miRNAs, the ten circRNAs most likely to sponge hsa-miR-30a and hsa-let-7b in BC were also identified (Figure 5E).

RNA Binding Proteins (RBPs) are crucial post-transcriptional regulators, and there are many RNA binding domains with which different RBPs can interact. Abnormal interactions between RBPs and RNA usually occur during cancer development. Among the more than 100 possible RNA modifications, m6A modification is one of the most common and is closely related to pathogenesis in multiple tumors as well as to drug responses [16]. RMBase was used to identify the top 20 RBPs that might interact with m6A modifications in SPC25 mRNA (Table 1).

**Table 1:** Top 20 relationships between m6A RNA modification and RBP binding regions in human.

**Gene Name**	**RBP Name**	**RBP ID**	**Mod ID**	**Support Num**	**Region**
SPC25	BUD13	RBP_site_1701016	m6A_site_273437	17	intron, utr5, intron
SPC25	DDX3X	RBP_site_1701017	m6A_site_273437	17	intron, utr5, intron
SPC25	EIF4A3	RBP_site_1701018	m6A_site_273437	17	intron, utr5, intron
SPC25	EIF4G2	RBP_site_1701019	m6A_site_273437	17	intron, utr5, intran
SPC25	ELAVL1	RBP_site_1701020	m6A_site_273437	17	intron, utr5, intron
SPC25	FBL	RBP_site_1701021	m6A_site_273437	17	intron, utr5, intron
SPC25	FMR1	RBP_site_1701022	m6A_site_273437	17	intron, utr5, intron
SPC25	FXR2	RBP_site_1701023	m6A_site_273437	17	intron, utr5, intron
SPC25	HNRNPM	RBP_site_1701024	m6A_site_273437	17	intron, utr5, intron
SPC25	IGF2BP2	RBP_site_1701025	m6A_site_273437	17	intron, utr5, intron
SPC25	LARP7	RBP_site_1701026	m6A_site_273437	17	intron, utr5, intron
SPC25	NOP58	RBP_site_1701027	m6A_site_273437	17	intron, utr5, intron
SPC25	SF3A3	RBP_site_1701028	m6A_site_273437	17	intron, utr5, intron
SPC25	SRSF1	RBP_site_1701029	m6A_site_273437	17	intron, utr5, intron
SPC25	SRSF9	RBP_site_1701030	m6A_site_273437	17	intron, utr5, intron
SPC25	AGO	RBP_site_1701007	m6A_site_273436	14	cds, exon
SPC25	DDX3X	RBP_site_1701008	m6A_site_273436	14	cds, exon
SPC25	EIF4A3	RBP_site_1701009	m6A_site_273436	14	cds, exon
SPC25	ELAVL1	RBP_site_1701010	m6A_site_273436	14	cds, exon
SPC25	FBL	RBP_site_1701011	m6A_site_273436	14	cds, exon

#### Genes and proteins co-expressed with SPC25 are associated with cell cycle progression and DNA replication and repair

**Figure 6:**
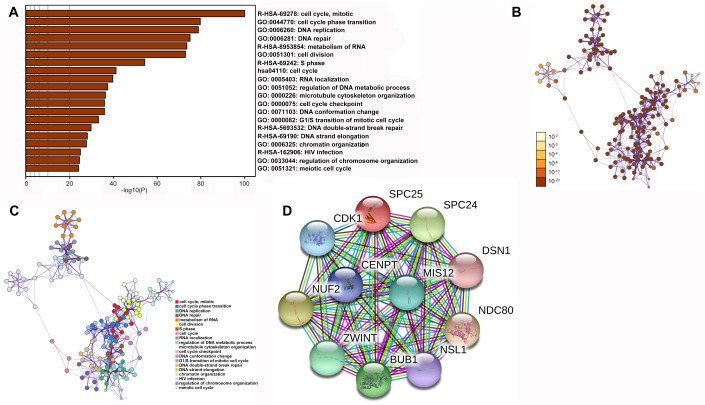
**Functional enrichments and protein interactions of SPC25.** (**A**) Results of KEGG analysis of genes co-expressed with SPC25. (**B**) The network of enriched terms colored by p-value; terms containing more genes tend to have smaller p-values. (**C**) The network of enriched terms colored by cluster ID; nodes that share the same cluster ID are typically close to each other. (**D**) Interactions between SPC25 and other proteins.

Enrichment analysis of co-expression genes performed using Metascape indicated that SPC25 is primarily involved cell cycle progression and DNA replication and repair processes (Figure 6A–6C), which is consistent with the single-cell analysis results. A STRING interactive network was used to identify proteins which can bind directly to SPC25 (Figure 6D).

### *SPC25* expression was correlated with immune factors

**Figure 7:**
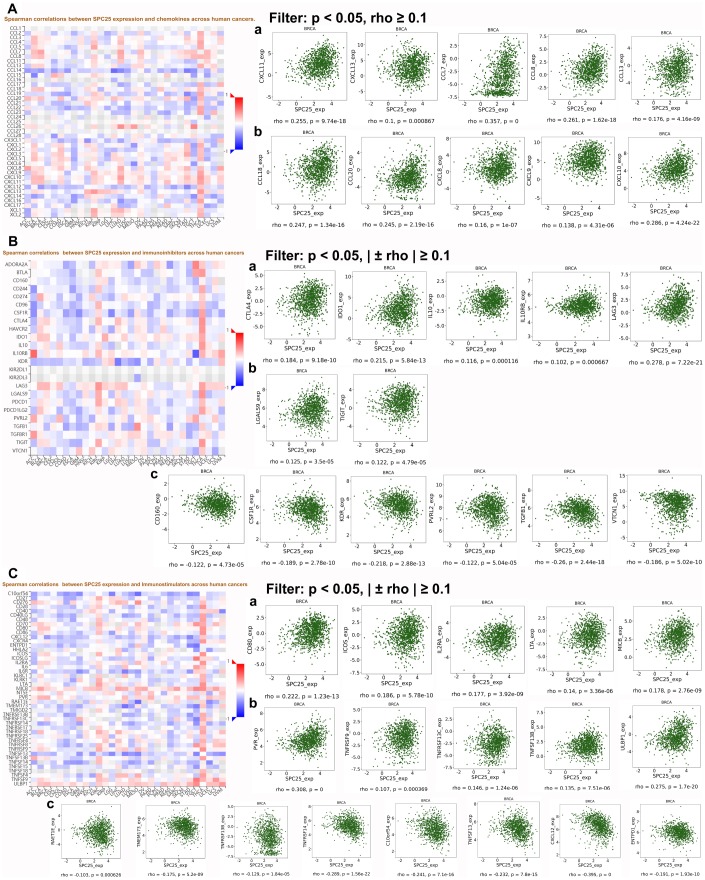
**Correlations between SPC25 expression and three cancer-related immune factor types.** (**A**) Correlation between SPC25 and chemokines in BC. (**B**) Correlation between SPC25 and immunoinhibitors in BC. (**C**) Correlation between SPC25 and immunostimulators in BC.

Growing evidence indicates that the immune system is closely to tumor development and progression. We therefore investigated the relationship between *SPC25* expression and immune factors; chemokines, immunoinhibitors, and immunostimulants for which expression was strongly correlated with *SPC25* expression after filtering for p < 0.05 and |±rho| ≥ 0.1 are shown in Figure 7. All databases used for analyses in this article are listed in Table 2.

**Table 2:** Summary of databases used in this study.

**Name**	**Link**	**This study**	**Keywords**
**TCGAportal**	http://www.tcgaportal.org	To investigate the expression of SPC25 in human tissues	gene expression; 28 cancer types; survival curve; DNA methylation; mutation
**FIREBROWSE**	http://firebrowse.org/		gene expression profile analysis profile
**UALCAN**	http://ualcan.path.uab.edu/	To analyze the SPC25 mRNA expression in different races, ages, molecular subtypes of BC patients	gene expression; 16 cancer types; survival curve; DNA methylation
**Human Protein Atlas**	https://www.proteinatlas.org/	To detect SPC25’s distribution and subcellular localization	proteins distribution; subcellular localization; impact for survival
**CANCERTOOL**	http://web.bioinformatics.cicbiogune.es/CANCERTOOL/index.html	To explore the relationship between SPC25 expression and relapse in BC	4 cancer types gene expression; genes correlation; functions and processes
**Kaplan-Meier Plotter**	http://kmplot.com/analysis/index.php?p=background	To analyzed the correlations between SPC25 mRNA expression and DMF, RFS as well as OS	breast cancer; subtype; survival curve;
**CancerSEA**	http://biocc.hrbmu.edu.cn/CancerSEA/	To observe the potential roles of SPC25 in BC	41,900 single cells 25 cancer types; 14 functional states;
**Cistrome DB Toolkit**	http://dbtoolkit.cistrome.org	Selected the TF of SPC25 - FOXM1	transcription factor; histone modifications
**MEXPRESS**	https://mexpress.be/	To unearth the methylation details of SPC25 as well as the relationship between SPC25 mRNA expression and different clinical characteristics of BC	gene expression; 34 cancer types; DNA methylation
**GEPIA 2**	http://gepia2.cancer-pku.cn/#index	To assess the correlations between genes	gene expression; survival curve; isoform details; genes correlation; similar genes detection
**LinkedOmics**	http://www.linkedomics.org/admin.php	To predict the miRNAs related to SPC25	32 cancer types; mRNA / protein expression; target genes; enrichment analysis
**TargetScanHuman**	http://www.targetscan.org/vert_71/		gene; miRNA
**starBase v3.0**	http://starbase.sysu.edu.cn/index.php	To predict the miRNAs related to SPC25; To perform circRNAs prediction, survival analysis of miRNAs as well as correlation analysis between miRNAs and SPC25 mRNA	miRNA target; ceRNA network; RBP target; RBP motif; Pathway; Pan-cancer
**Metascape**	http://metascape.org/gp/index.html#/main/step1	To obtained the heatmap and network of enrichment terms related to SPC25	gene annotation; membership search; functional enrichment; interactome analysis
**STRING**	https://string-db.org/cgi/input.pl	To obtain the interaction network between SPC25 and other important proteins	protein details; interactive network; functional enrichment
**DISIDB**	http://cis.hku.hk/TISIDB/index.php	To analyze the correlations between expressions of SPC25 mRNA and 3 kinds of immune factors	immune system; 30 cancer types; immunotherapy
**RMBase v2.0**	http://rna.sysu.edu.cn/rmbase/index.php	To predict potential RBP of SPC25 mRNA	RNA modification; RBP prediction; RNA - miRNA binding

## DISCUSSION

In a previous study, *SPC25* expression was higher in basal breast cancer subtypes compared with other subtypes, suggesting that it might play a key role in basal stem cell-driven breast cancer [9]. In this study, we investigated *SPC25* expression in breast cancer (BC) and in various other human cancer types and found that SPC25 expression was upregulated in multiple tumors. To further examine the effects and predictive value of elevated SPC25 levels, we performed an analysis using CancerSEA. The results indicated that SPC25 might influence BC development and progression by regulating the cell cycle, DNA damage and repair, and cell proliferation.

Colocalization of SPC25 and microtubule proteins has been observed within cells. Previous studies have demonstrated that tubulin is crucial in the cell cycle and cell proliferation [17]. Furthermore, Kostrhunova *et* *al*. found that a subset of new platinum antitumor agents kills cells in part by altering microtubule cytoskeleton organization [18]. It is therefore possible that SPC25 might promote BC development and progression by binding to microtubule proteins. Future studies should investigate whether SPC25 is associated or colocalized with microtubule proteins in BC cells.

Notable DNA methylation modifications were identified in the SPC25 gene, and its expression was positively correlated with *DNMT* expression. Topper *et* *al*. designed a new sequential low-dose regimen that proved very effective in treating non-small-cell lung cancer (NSCLC) by investigating pharmacologic and isotype specificity of histone deacetylase inhibitors (HDACis) and their interactions with DNA-demethylating agents (DNA methyltransferase inhibitors [DNMTis]) [19]. 5-Aza-2′-deoxycytidineon (5-Aza-CdR) is an effective demethylation agent that can block cell cycle progression, induce apoptosis, promote differentiation, and reduce invasion and metastasis in tumor cells, ultimately inhibiting their growth. Our results suggest that BC development and progression might be mediated by alterations in DNA modifications of the SPC25 gene; 5-Aza-CdR might therefore be an effective treatment for BC patients.

Maternal embryonic leucine zipper kinase (MELK) is a novel oncogene that plays a crucial role in the TP53 pathway, and OTSSP167 is an oral medication that inhibits MELK. Previous studies have demonstrated that MELK inhibition promotes cancer cell death through the p53 signaling pathway. Zhang *et* *al*. reported that MELK might be an effective therapeutic target in CLL, and OTSSP167 exhibited potent anti-tumor activities in CLL cells [20]. Here, we confirmed that TP53 gene expression was positively correlated with SPC25 expression, suggesting that OTSSP167 might also inhibit SPC25 expression by suppressing the TP53 pathway. Combined treatment with 5-Aza-CdR and OTSSP167 might therefore be particularly effective in BC patients, although additional research is necessary to elucidate the molecular mechanisms underlining their effects. In addition, due to the association between FOXM1 and SPC25 in BC, TF inhibitors might also help to improve BC treatments and prognosis.

Interactions between tumors and immune cells are a growing subject of research, and many studies are currently evaluating the clinical value of immunomodulation in BC patients. Hammerl *et* *al*. suggested that the quantity and quality of tumor-infiltrating lymphocytes (TILs) differs among BC subtypes, as does the occurrence and antigenicity of immune evasion mechanisms [21]. All of these factors should therefore be evaluated when designing and selecting the best immunotherapy for specific BC patient subgroups. DISIDB results indicated that *SPC25* expression was lowest in C3 (inflammatory) and highest in C2 (IFN-γ dominant) immune cell subtypes. More importantly, we identified associations between many chemokines and SPC25 expression in BC. For example, CCL7 (rho=0.357, p=0) is derived from cancer-associated fibroblasts (CAFs) in the interstitial microenvironment. Studies have shown that CCL7 promotes migration and invasion of cancer cells in a dose-dependent manner [22]. Knockdown of SPC25 inhibited the proliferation of BC cells, and SPC25 expression was positively correlated with CCL7. It is therefore possible that SPC25 is a downstream target of CCL7 and is partially responsible for CCL7-induced promotion of tumor cell proliferation. In addition, we found that some immunological checkpoints that have recently received increased attention, such as CXCL8, CTLA4, IDO1, and TIGIT, are positively correlated with *SPC25* expression in BC. It is possible that these immune checkpoints are expressed at even higher levels in TNBC, in which SPC25 expression is also higher, than in other BC subtypes. Inhibitors targeting these immunological checkpoints might therefore be particularly effective in TNBC patients, especially in combination with SPC25 inhibitors such as OTSSP167. Furthermore, this higher *SPC25* expression might explain the poorer prognosis and shorter survival times observed in TNBC patients. Finally, the immunostimulant CXCL12 is negatively correlated with SPC25; if SPC25 expression is an effective indicator of prognosis in BC, CXCL12 might also serve as a useful immunotherapy biomarker by reflecting the status of the immune microenvironment in BC patients.

Together, these findings suggest that *SPC25* expression, which is highest in BC subtypes with the poorest prognoses, is associated with survival in BC patients. In addition, some miRNAs and circRNAs may promote BC development and progression by upregulating SPC25 mRNA levels. It is therefore of great clinical importance to investigate DNA methyltransferase inhibitors and TF inhibitors that might downregulate SPC25. SPC25 might also serve as a biomarker for evaluating the status of the tumor microenvironment and as an immunotherapy target in BC patients.

## MATERIALS AND METHODS

### SPC25 expression level analysis

TCGAportal (http://www.tcgaportal.org) and FIREBROWSE (http://firebrowse.org/) were used to investigate the expression of SPC25 in different tumor tissues and corresponding para-carcinoma tissues. The Human Protein Atlas (https://www.proteinatlas.org/) database contains pathology and gene information from many reports on various tissues and cells. We used it to examine SPC25 expression in different tissues and the location of SPC25 mRNA within cells. Next, we compared SPC25 expression in BC patients of different races, ages, and molecular subtypes using UALCAN (http://ualcan.path.uab.edu/). The Wilcoxon rank sum test was used to assess the significance of observed differences.

### Relapse and survival analysis

CANCERTOOL (http://web.bioinformatics.cicbiogune.es/CANCERTOOL/index.html) is an open-access resource for the analysis of gene expression and functional enrichments in BC, colorectal cancer, lung cancer, and prostate cancer [23]. Here, it was used to explore the relationship between SPC25 expression and relapse in BC. Student’s t-test was used to compare mean gene expression between two groups. We then used Kaplan-Meier Plotter (http://kmplot.com/analysis/index.php?p=background), an online survival analysis tool containing data on the effects of 22,277 genes on breast cancer prognosis based on 1809 patient microarrays [24], to examine correlations between *SPC25* expression and DMF, RFS, and OS. The two patient cohorts were compared using a Kaplan-Meier survival plot, and hazard ratios with 95% confidence intervals and log rank p-values were calculated.

### Single-cell analysis

We used CancerSEA (http://biocc.hrbmu.edu.cn/CancerSEA/), which depicts single-cell functional status maps that include 14 functional states for 41,900 individual cells from 25 cancer types [25], to explore the potential roles of SPC25 in BC.

### TF identification

The Cistrome DB Toolkit database (http://dbtoolkit.cistrome.org) allows users to query transcription factors (TFs) that might regulate genes of interest to identify binding factors, histone modifications, and chromatin accessibility in a genomic interval of interest up to 2 Mb in length. ChIP-seq, DNase-seq, and ATAC-seq samples with the most similarities are determined based on overlap with the user-provided genomic interval sets [26, 27]. We used the Cistrome DB Toolkit to predict which TFs are most likely to increase SPC25 expression in BC.

### DNA methylation modification analysis

MEXPRESS (https://mexpress.be/) is a data visualization tool for TCGA expression, DNA methylation status, clinical data, and the relationships between them [28]. Here, MEXPRESS was used to investigate methylation status of the SPC25 gene as well as the relationship between SPC25 mRNA expression and different clinical characteristics in BC patients.

### Gene correlations analysis

GEPIA2 (http://gepia2.cancer-pku.cn/#index) is an open-access dataset for analyzing RNA sequencing expression data from 9,736 tumors and 8,587 normal samples from the TCGA and GTEx projects. It provides tumor/normal differential expression analysis, profiling according to cancer types or pathological stages, patient survival analysis, similar gene detection, correlation analysis, and dimensionality reduction analysis. GEPIA2 was used throughout this study to assess correlations between all important genes.

### Identification of miRNAs and circRNAs that target SPC25

LinkedOmics (http://www.linkedomics.org/admin.php) allows for analysis of multi-omics data within and across 32 cancer types. TargetScanHuman (http://www.targetscan.org/vert_71/) can predict biological targets of miRNAs by searching for the presence of conserved 8mer, 7mer, and 6mer sites that match the seed region of each miRNA (Lewis et al., 2005). starBase v3.0 (http://starbase.sysu.edu.cn/index.php) is an open-source platform for the identification of miRNA-ncRNA, miRNA-mRNA, ncRNA-RNA, RNA-RNA, RBP-ncRNA, and RBP-mRNA interactions from CLIP-seq, degradome-seq, and RNA-RNA interactome data. We used these three databases to identify miRNAs that are very likely to bind to SPC25 mRNA. In addition, starBase v3.0 was used to perform circRNA prediction, miRNA survival analysis, and analysis of correlations between miRNAs and SPC25 mRNA.

### Protein-protein interaction and functional enrichment analysis

Metascape (http://metascape.org/gp/index.html#/main/step1) is a web-based portal that combines functional enrichment, interactome analysis, gene annotation, and membership search using data from more than 40 independent knowledgebases. In addition, it facilitates comparative analysis of multiple independent and orthogonal experiments across datasets [29]. Metascape. STRING (https://string-db.org/cgi/input.pl) is a database of known and predicted protein-protein interactions including direct (physical) and indirect (functional) associations; these interactions are derived from computational predictions, knowledge transfer between organisms, and interactions aggregated from other (primary) databases [30]. We utilized STRING to create an interaction network between SPC25 and other important proteins.

### Immune-related analysis

DISIDB (http://cis.hku.hk/TISIDB/index.php) is a web portal that integrates multiple heterogeneous data types for analysis of tumor and immune system interactions [31]. Here, it was used to analyze Spearman correlations between SPC25 expression, two types of immunomodulators, and chemokines.

### Analysis of SPC25 mRNA mutation and potential RBP

TCGAportal (http://www.tcgaportal.org), an online portal that allows for parallel alignment of multiple tumors as well as detailed analysis of individual tumors, was used for mutation analyses of SPC25 mRNA. RMBase v2.0 (http://rna.sysu.edu.cn/rmbase/index.php) is a web tool for RNA-level epigenetic modification queries using data from both CeU-seq in the GEO database and the Sequence Read Archive (SRA) database. In addition to m6A, it can also query RNA and miRNA binding, RNA-binding protein molecules, etc. Here, potential interactions between RBPs and SPC25 mRNA were predicted using RMBase.

### Cell culture and transfection

All cells were cultured using RPMI-1640 Medium (Gibco, USA) supplemented with 10% fetal bovine serum (FBS) (YEASEN, China) at 37°C in a 5% CO_2_ chamber with penicillin (100 IU/mL) and streptomycin (100 mg/mL). Small interfering RNA against SPC25 (si-SPC25) and non-targeting control siRNA (si-NC) were synthesized by RiboBio Company (Guangzhou, China). Transfections were performed with Lipofectamine 2000 (Invitrogen, USA) and Opti-MEM (Gibco, USA). Cells were harvested 48 h post-transfection for further study. The target sequence of si-SPC25 was as follows: 5′- GGUGAGAAAUUGCAGUUUAUU -3′.

### RNA extraction and quantitative real-time polymerase chain reaction (qRT-PCR)

Total RNA was isolated using an RNA extraction kit (TIANGEN, China) according to the manufacturer’s instructions. qRT-PCR was performed to measure gene expression using the Hieff® qPCR SYBR Green Master Mix kit (YEASHEN, China). Glyceraldehyde 3-phosphate dehydrogenase (GAPDH) was used as an internal control. Results were analyzed using the 2−ΔΔCt method. Primer pairs for SPC25 were as follows: 5′- AGTACGGACACCTCCTGTCAG-3′ (Forward) and 5′- TCTCAACCATTCGTTCTTCTTCC -3′ (Reverse).

### Cell proliferation experiments

For the colony formation assay, MDA-MB-231 and MCF-7 cells were transfected with either si-SPC25 or si-NC. After 10 days, cells were washed three times with cold PBS and fixed with 4% paraformaldehyde. Cells were then stained with crystal violet. Colony numbers in each well were counted and photographed.

For the Cell Counting Kit-8 (CCK-8) assay, cells were first transfected with either si-SPC25 or si-NC and incubated at 37°C. CCK-8 solution (Beyotime, China) was then added to each well and incubated for 2 h. Absorbance at 450 nm was measured at 0, 24, 48, 72, and 96 h timepoints.

### Statistical analysis

Data are shown as mean ± standard deviation (SD). Student’s t-test or one-way analysis of variance (ANOVA) performed in GraphPad Prism 7 were used for statistical analysis of all cell line experiments. Gene expression correlations were assessed using Spearman’s correlation. A p-value < 0.05 was considered statistically significant. Corresponding significance levels are presented in the figures.

## Supplementary Material

Supplementary Figure 1
