# Supplementary material for: Up-regulation of SPC25 promotes breast cancer
Source: Aging (Albany NY). 2019 Aug 10;11(15):5689–704. doi: 10.18632/aging.102153 (PMC6710047; doi:10.18632/aging.102153)
Supplement: Supplementary Figure 1 [file aging-11-102153-s001.pdf]

SUPPLEMENTARY FIGURE

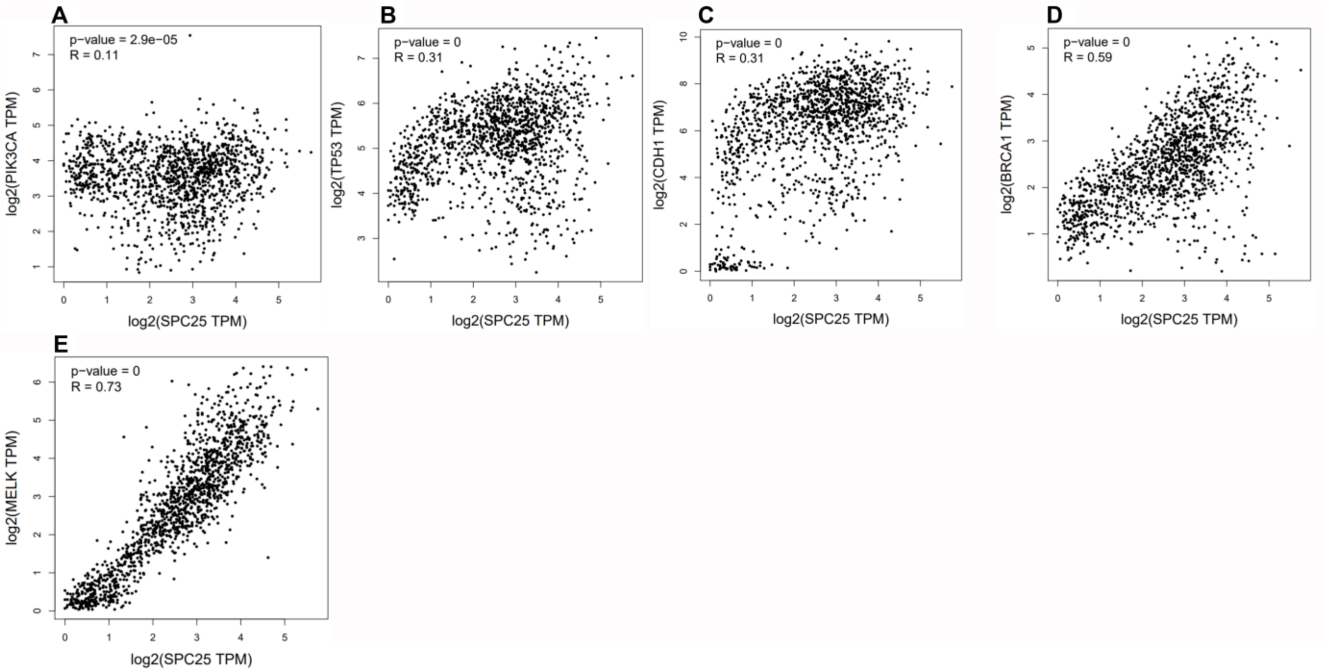

Supplementary Figure 1. Analysis of correlations between SPC25 and related important genes.
